# From vulnerability to duplicity: Examining the connection between childhood adversity and deception

**DOI:** 10.1371/journal.pone.0321666

**Published:** 2025-04-23

**Authors:** David M. Markowitz

**Affiliations:** Department of Communication, Michigan State University, East Lansing, Michigan United States of America; National University of Medical Sciences, PAKISTAN

## Abstract

Deception research has traditionally evaluated how individual differences like personality traits and demographics correlate with lying. However, the establishment of adverse childhood experiences (ACEs) as an individual difference that also links to deception remains underexplored. To this end, the present study (*N* = 784 students) investigated the relationship between ACEs and deception in adulthood. Results indicated that individuals with more (versus less) adverse childhood experiences, particularly those involving maltreatment and victimization, reported more daily white and big lies, independent of aversive personality traits like narcissism and Machiavellianism. Consistent with other studies on individual differences and deception, the effect sizes were small, but systematic. Together, these findings support the *dispositional honesty hypothesis*, indicating that foundational childhood experiences and events can shape or signal deceptive behavior. Generally, the study contributes to our underexamined knowledge base of the developmental antecedents of lying, emphasizing the role that adversity plays during childhood to influence deceptive behavior beyond commonly studied personality traits.

How often do people lie, and what characteristics betray a liar from a truth-teller? The deception literature has examined these questions for decades. Evidence overwhelmingly suggests most people are honest most of the time (e.g., reporting one-to-two lies per day), and there are a few people in a population who tend to lie more than the average person [1–10]. Indeed, lying rates are often not normally distributed, a result that is both pancultural and relatively stable across investigations [11,12]. Regarding *who* lies, there is comparatively less research in this empirical space relative to *how often* people lie. Studies suggest people who are young, male, high on aversive personality traits (i.e., Machiavellianism, narcissism, psychopathy), and those who believe others are frequent liars tend to lie more than average person [1,6,7,9,13]. Together, lying is a constrained yet purposeful act, and there are several enduring characteristics that identify those who tell many (versus few) lies in everyday life.

Deception research — like most subfields in the social sciences — has long debated the role of the person versus the situation in determining one’s proclivity to lie. As recent work indicates [14], some people are dispositionally honest (e.g., their psychological makeup is more likely to be honest, cooperative, and fair to others; [15]) and others are situationally honest (e.g., depending on the setting or situation, people may be more or less prone to honesty; [11,16,17]). Some people who are dispositionally honest can also be enticed by the situation to lie (e.g., when the truth is a problem, if there is an incentive). Both the person and the situation are plausible explanations for deception, though more work on the link between individual differences or dispositional characteristics and deception is needed to develop a profile of who tends to lie in society [6]. Against this backdrop, the present study seeks to expand the *dispositional honesty hypothesis* by identifying how largely unexamined individual differences, like adverse childhood experiences (ACEs), relate to deceptive behavior. Individual differences, which are enduring psychosocial characteristics that define an individual and distinguish one person from another (e.g., personality), are essential to investigate in deception research with the hope of building a profile of characteristics that indicate liars from truth-tellers. The point of this research is not to ignore or undervalue the role of the situation and its influence on dishonesty, but instead, to identify how relatively stable traits like one’s adverse childhood experiences might signal those who are inclined to lie versus tell the truth.

## Individual differences, adverse childhood experiences, and deception

The idea of investigating the connection between individual differences like adverse childhood experiences and deception is rooted in social learning theory [18,19]. At its core, the theory suggests people learn through observation and imitation in one’s environment. Learning is iterative and ongoing in that people symbolically understand, interpret, and reinforce what is learned in one setting, and apply it to other settings. Given that children from abusive or distressing homes tend to experience a different “communication environment” than children from non-abusive or non-distressing homes [20], it is reasonable that children learn how to trust (or not trust) and believe (or not believe) others at an early age [21], using parents or parental figures as models for these behaviors. Finally, other work suggests victimized and abused children are typically less trusting of others, which can lead to their greater detection accuracy of lies and truths [22]. Altogether, children are social learners from an early age and therefore, the present work examines how being victimized, mistreated, and less trusting of others during foundational periods of life might link to downstream deceptive tendencies.

The current work treats adverse childhood experiences — defined in early work as “childhood abuse and household dysfunction” [23] — as an individual difference (e.g., an enduring psychological characteristic) and connects this concept to deception, making it important to first briefly review other ways that individual differences and deception have been studied. The most common connection between individual differences and deception has been through the evaluation of personality traits and demographics. Prior work suggests people who are high on traits comprising The Dark Triad (i.e., Machiavellianism, narcissism, psychopathy) tend to cheat more in behavioral tasks [6,13,24] and self-report more daily lying [1]. Similarly, high sensation-seekers tend to cheat more than low sensation-seekers [25]. Regarding demographics, men tend to report more deceptive behavior than women across various settings [6,9,26,27], and younger individuals lie more than older individuals [28]. Together, individual differences like personality and demographics comprise a major focus of deception scholarship to understand the characteristics of liars versus truth-tellers. Less attention has focused on how early and foundational childhood experiences — particularly those that are negative and adverse in nature — relate to lying, which represents the primary interest of this work.

When children or childhood experiences are the focus of deception research, however, much work has attended to how children lie and when they develop the capacity for deception. Children understand the social and psychological consequences of deception at around 3 years old, and 3–7-year-olds reliably tell white lies (e.g., socially acceptable lies that have little-to-no consequences for the lie recipient; [29]) out of politeness concerns [30,31]. Crucially, according to prior work [32], children learn about lying from social-environmental influences. Chief among these influences are parents, who serve as “the primary social agents in children’s early lives” and impact what children view as acceptable or unacceptable behavior [32].

What specific influence can parents and early childhood experiences have on lying? Talwar and Crossman [32] identify that parents may have direct and indirect influences on their children’s deception. For example, teaching children about honesty through stories can demonstrate the value of truth-telling and promote honesty [33,34]. Parents who have allowed, minimized, or ignored lying by their children were also more likely to have children who were prone to deceive nearly two years later [35]. Therefore, deception can become an absorbed or accepted behavior based on how children directly learn from or become socialized by their parents. Indirectly, parents can influence their children’s lying behavior as well. In a self-control and temptation-resistance study, children who were lied to were also more likely to lie in a subsequent cheating task [36]. This type of social learning and modeling has been reported in other settings as well [37].

Taken together, prior evidence suggests various individual differences (e.g., aversive personality traits, demographics) that relate to one’s deceptive tendencies, and experiences that children have with their parents can directly and indirectly influence their downstream behavior. Put another way, “the combination of social-environmental influences with individuals’ own characteristics and skills, interacting across situations, over time, shape trajectories of lie-telling across the lifespan” [32]. The present study attempts to systematically evaluate the relationship between ACEs and deception to test how early and foundational events during one’s upbringing associate with their present-day behavior.

Despite one qualitative inquiry on fake identities that has attempted to link ACEs and deception [38], it remains unclear how early adverse childhood experiences (e.g., parents who engaged in risky behavior, parents who were absent, neglectful experiences during adolescence), might relate to one’s deceptive tendencies in adulthood. This empirical uncertainty represents the core interest of the current work and thus, the following research question is proposed:

RQ: What is the relationship between ACEs and individual differences (e.g., aversive personality traits), lie production, and deceptive behavior?

## Method

### Power and participants

An *a priori* power analysis estimating a small effect (*r* =.10) at 80% power (α =.05, two-tailed) suggested 782 participants were required for this study. A total of 784 participants were recruited from the Department of Communication’s participant pool at Michigan State University. This study, which ran from 3/25/2024–9/22/2024, was approved by the Michigan State University Institutional Review Board (STUDY00010314) and written/typed informed consent was obtained.

On average, participants were 19.79 years old (*SD* = 1.85 years). Most identified as women (*n* = 466, 59.4%; men *n* = 313; other *n* = 3) and White (*n* = 625, 79.7%; Asian *n* = 75; Black or African American *n* = 46; Other *n* = 31; Native Hawaiian or Pacific Islander *n* = 3; American Indian or Alaska Native *n* = 2). Regarding political ideology, participants were “middle of the road” on a 7-point scale, on average (*M* = 4.09, *SD* = 1.46).

### Procedure

To evaluate the relationship between ACEs and deception, participants provided written/typed informed consent and then were introduced to the Adverse Childhood Experiences Scale [23]. Next, participants were introduced to several self-reported lie production measures, including how many times they tell a white and big lie each day on average, plus how many times they believe others tell a white and big lie per day. White lies were conceptually defined as socially acceptable lies that have little-to-no consequences for the lie recipient [29] and big lies are serious lies that have great “cognitive and emotional significance” [2]. Participants then completed a behavioral measure of cheating adopted from prior work [6,39,40]. Specifically, participants were told to unscramble letters to form eight words in English and indicate those that they were able to solve, though three trials were unsolvable and counted as cheating. Finally, participants provided responses to aversive personality trait measures (i.e., Machiavellianism, narcissism, psychopathy) along with demographics. Participants were debriefed and then exited the survey.

### Measures

#### Adverse childhood experiences scale.

ACEs were measured with the 10-item Adverse Childhood Experiences scale [23]. This scale asks participants if they experienced various harms in their youth (e.g., if they had parents who may have insulted or humiliated them, if they had parents who were incarcerated). People with more ACEs typically experience more mood and anxiety disorders [41,42], suicide attempts [43], or risky behavior like illicit drug use [44] than people with less ACEs. Responses to each item were binary (1 = yes, 0 = no), and items were summed to create a composite (min = 0, max = 10). High numbers suggest a person had more adverse childhood experiences than low numbers.

The 10 items typically load onto two factors: (1) child maltreatment and peer victimization, and (2) household challenges [45]. Indeed, an exploratory factor analysis confirmed a two-factor solution that accounted for 43.7% of the variance. The ACEs scale was evaluated as a collection of 10 items and at the item level for completeness.

#### Self-reported lie production.

Consistent with prior work [6,8,9,39], participants self-reported how often they lied in a typical day and how often they believed others lie in a typical day. To ground participants in the topic of deception, the following instructions were provided:

*Most people think a lie occurs any time you intentionally try to mislead someone. Some lies are big while others are small; some are completely false statements and others are truths with a few essential details made up or left out. Some lies are obvious, and some are very subtle. Some lies are told for a good reason. Some lies are selfish; other lies protect others. Below, we are interested in all these different types of lies*.

Then, participants reported their daily lying rates (“On average, how many times a day do you tell a [little white/big] lie?”), and their perceptions of others’ daily lying rates (“On average, how many times a day do you think other people tell a [little white/big] lie?”), with 10 response options (0, 1, 2, 3, 4, 5, 10, 15, 20, 25+ lies). All 25+ lies were recoded as 25.

#### Deceptive behavior (Cheating).

Participants were presented with 8 anagrams (word jumbles) to solve in two minutes, and they were required to unscramble the jumbles to form words in English. Five trials were solvable (i.e., TTSIRA, SREETD, LONSEM, EESPRMU, TTEDES), and three were unsolvable (i.e., OPOER, ALVNO, ANDHU). Participants who claimed to solve an anagram clicked a “solved” button next to the word jumble and if they could not, they checked “unsolved.” Blank responses were counted as “unsolved.” Scores on the three unsolvable trials counted as cheating and were summed to a deceptive behavior score (min = 0, max = 3).

#### The Dark Triad.

To evaluate how individual differences like ACEs associate with deception, independent of other individual differences known to associate with lying, three indicators of The Dark Triad were assessed [13,46]. Narcissism describes one’s inflated sense of self-importance and a need for admiration, Machiavellianism describes one’s tendency to manipulate and exploit others, and psychopathy describes those who lack of empathy and high on impulsivity. The 27-item questionnaire was measured using 5-point Likert type scale agreement questions (1 = strongly disagree, 5 = strongly agree). Items within each 9-item subscale were averaged and reliable as collections (Machiavellianism Cronbach’s α = 0.72; narcissism Cronbach’s α = 0.67; psychopathy Cronbach’s α = 0.73).

The paper’s minimal dataset is available on the Open Science Framework: https://osf.io/u2b4p/.

## Results

### Descriptive patterns

**Fig 1:**
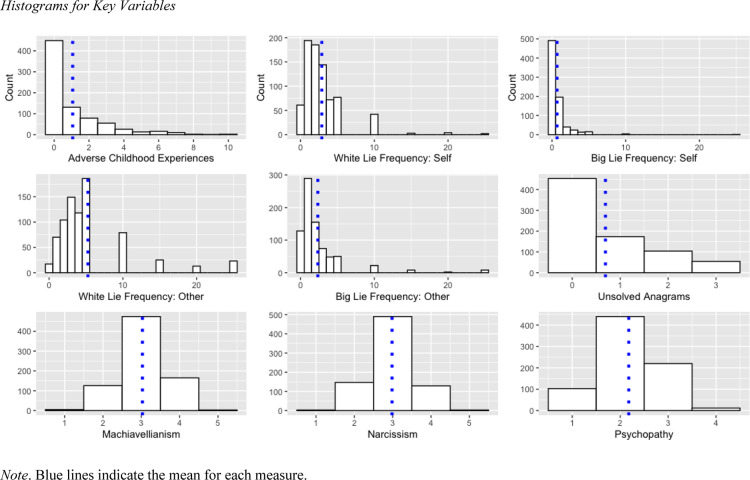
Histograms for Key Variables. *Note*. Blue lines indicate the mean for each measure.

As expected based on prior work [6,11,47], lying rates were not normally distributed (Fig 1), and most participants reported telling only a few lies per day. On average, participants told 2.89 white lies per day (*SD* = 2.88 white lies) and 0.69 big lies per day (*SD* = 1.52 big lies).

Consistent with prior work as well [6,39], self-reported big lies were positively associated with behavioral deception or cheating (ρ =.149, *p* <.001). Self-reported white and big lying rates were positively correlated with other-perceived white and big lying rates (ρs >.47, *p*s <.001), evidence supporting the deception consensus effect [6,7,39,48]. Regarding lying differences by demographics, men and women differed on self-reported big lying rates (Welch’s *t*(444.51) = 2.87, *p* =.004, Cohen’s *d* = 0.22), with men reporting more daily big lies than women. Men also cheated more on the behavioral anagram task than women (Welch’s *t*(581.02) = 2.44, *p* =.015, Cohen’s *d* = 0.18). Other relationships between deceptive behavior and demographics were not statistically significant at the 5% level.

### Links between adverse childhood experiences and deception

The evidence in Table 1 suggests participants who reported more ACEs also told more white and big lies than people who reported less ACEs. People who reported more ACEs also believed that others told more white lies than people who reported less ACEs. Regarding aversive personality traits, ACEs were positively associated with Machiavellianism and psychopathy, but negatively associated with narcissism. Finally, ACEs and cheating behavior were not statistically associated at the 5% level. Intercorrelations between key variables are represented in Table 1 for transparency and to identify how such dimensions are generally related.

**Table 1:** Correlation Matrix Between Key Variables.

Number	Spearman’s rho	1	2	3	4	5	6	7	8	9
1	ACES	--								
2	Self: White lying	**.11**	--							
3	Self: Big lying	**.07**	**.53**	--						
4	Other: White lying	**.10**	**.63**	**.36**	--					
5	Other: Big lying	.05	**.42**	**.47**	**.63**	--				
6	Unsolved anagrams	-.06	.03	**.15**	**.07**	**.17**	--			
7	Machiavellianism	**.11**	**.26**	**.21**	**.16**	**.14**	.06	--		
8	Narcissism	**-.07**	.01	.03	.02	**.12**	**.13**	**.19**	--	
9	Psychopathy	**.07**	**.20**	**.27**	**.11**	**.19**	**.10**	**.48**	**.25**	--
Number	Significance values	1	2	3	4	5	6	7	8	9
1	ACES	--								
2	Self: White lying	.00	--							
3	Self: Big lying	.04	.00	--						
4	Other: White lying	.00	.00	.00	--					
5	Other: Big lying	.20	.00	.00	.00	--				
6	Unsolved anagrams	.08	.42	.00	.04	.00	--			
7	Machiavellianism	.00	.00	.00	.00	.00	.11	--		
8	Narcissism	.05	.71	.43	.60	.00	.00	.00	--	
9	Psychopathy	.04	.00	.00	.00	.00	.00	.00	.00	--

*Note*. Bolded correlations in the top panel are statistically significant at *p* <.05.

Regarding individual items of the ACEs scale, self-reported white lying was mostly associated with items of child maltreatment and peer victimization (compared to household challenges). That is, self-reported white lying was positively associated with parents insulting or swearing at children (ρ =.156, *p* <.001), physical abuse like parents pushing, grabbing, or hitting children (ρ =.102, *p* =.004), the feeling that no one in their family love them or thought they were special (ρ =.103, *p* =.004), a motherly figure being physically abused like being pushed, grabbed, or slapped (ρ =.088, *p* =.014), and a household member being mentally ill (ρ =.108, *p* =.002). Self-reported big lying was positively associated with parents insulting or swearing at children (ρ =.106, *p* =.003), physical abuse like parents pushing, grabbing, or hitting children (ρ =.100, *p* =.005), the feeling that no one in their family love them or thought they were special (ρ =.091, *p* =.011), the feeling of not having enough resources or protection (ρ =.071, *p* =.045), a motherly figure being physically abused like being pushed, grabbed, or slapped (ρ =.071, *p* =.046), and a household member being mentally ill (ρ =.080, *p* =.025). As this evidence suggests, ACE indicators of deception were remarkably stable and consistent across white and big lies, and focused primarily on maltreatment and victimization.

Using partial Spearman correlations, the relationship between ACEs and white lying remained significant after controlling for Machiavellianism (ρ =.088, *p* =.014), narcissism (ρ =.110, *p* =.002), and psychopathy (ρ =.099, *p* =.006). The link between ACEs and big lying remained significant or at least marginally significant after controlling for Machiavellianism (ρ =.061, *p* =.089) and narcissism (ρ =.075, *p* =.038). The relationship between ACEs and big lying was not statistically significant after controlling for psychopathy (ρ =.056, *p* =.121).

## Discussion

The present study investigated the underexplored connection between adverse childhood experiences and downstream deceptive behavior. The results support the idea of adverse childhood experiences as an individual difference that systematically link to lying rates, independent of other individual differences that ordinarily associate with deception like aversive personality traits. The adverse childhood experiences most strongly connected to lying were childhood maltreatment and victimization. Crucially, therefore, specific childhood harms are linked to deception over others, providing theoretical scaffolding to understand how foundational and early experiences relate to how often people lie (or suggest that they lie).

This study makes several important contributions to deception research. First, it extends the *dispositional honesty hypothesis* [14] by identifying ACEs as a previously underexplored individual difference that relates to deceptive behavior. This finding adds nuance to our understanding of the person-situation debate or conversation in deception research, suggesting that early life experiences may inform or shape downstream dishonesty. Second, the study provides empirical support for the idea that different types of ACEs may have varying impacts on deceptive tendencies, with childhood maltreatment and victimization showing stronger associations with lying than household challenges. This distinction helps to refine how we think about early experiences and their influence on downstream deceptive behavior. It is unclear why childhood maltreatment and victimization associated with lying more so than household challenges, though some clinical research suggests experiences with neglect and abuse are antecedents to deception as well [49], evidence that complements the current empirical package. Future research would benefit from further investigation of the underlying explanations associated with differential ACEs and how they uniquely associate with deception.

More broadly, this work advances our understanding of individual differences and deception by demonstrating a link between ACEs and lying rates, independent of other known correlates like aversive personality traits. Therefore, this study reveals a new dimension in the profile of characteristics that may indicate who lies. Perhaps, some roots of deceptive behavior can be traced back to early life experiences, particularly those involving maltreatment and victimization. While the effect sizes in this study are small, they are consistent in size with others that appear in deception research [50,51], suggesting ACEs might have some diagnostic utility in identifying liars versus truth-tellers compared to other individual differences that are presently investigated in the literature. Indeed, the insights from this paper encourage deception scholars to consider a more comprehensive developmental perspective when examining individual differences in deception. Much research has investigated the antecedents and consequences of children lying [32,52], and less effort has focused on early childhood experiences as an indicator of future lying. Together, lying is not solely determined by immediate situational factors or personality traits, but may also be shaped by developmental experiences.

Finally, practical implications of this work include possible applications for mental health professionals or counselors who may be dealing with children (or adults) struggling with dishonesty [53]. That is, understanding the link between ACEs and deceptive behavior could inform therapeutic approaches for individuals struggling with honesty issues. Interventions that address underlying adverse childhood experiences may be more effective in promoting honesty than those focused on immediate behavior change. Second, in educational settings, this knowledge could guide the development of more compassionate and effective strategies for addressing when students lie, particularly those who have negative or at-risk home experiences. Rather than applying punitive measures, schools might implement supportive interventions that address some of the root causes of deceptive behavior, which could be maltreatment and victimization in the home.

Altogether, this research emphasizes the far-reaching consequences of adverse childhood experiences beyond common outcomes like mental health issues and substance abuse [54,55]. These consequences also include interpersonal behaviors like deception and lying. This underscores the critical importance of understanding deeply-rooted antecedents of deception to help understand how dispositional traits are indicative of those with deceptive tendencies.

## Limitations and future directions

There are several limitations of this work that require consideration. First, the results reported here are not direct cause and effect. Longitudinal research that tracks participants over time — perhaps, a multi-decade study that tracks infants through adulthood in terms of their childhood experiences and deception — might be informative to address this question. Second, the effect sizes are notably small but systematic. Most effect sizes reported here are within the range of those in deception research more broadly, however, and they add to our existing understanding of how certain individual differences are associated with deception at a broad theoretical level. Third, for the deceptive behavior measure, this was a one-shot instantiation of cheating. It is unclear if people who cheat one day (or on one task) would cheat on another day or a different task [56]. Further, this measure was relatively narrow in score and might not reflect general lying behavior across contexts. Evaluating adverse childhood experiences with other forms of deceptive behavior and cheating is advised. It is also important to note that the sample was limited to students, which is a concern for generalizability of the findings. Future research should attempt to evaluate these patterns among different, more representative populations and from those with varying socioeconomic backgrounds.
